# Fetal hypertrophic cardiomyopathy with elevated middle cerebral artery peak systolic velocity (MCA PSV): A potentially grim association

**DOI:** 10.1002/ccr3.7270

**Published:** 2023-04-23

**Authors:** Nathan A. Keller, Luis A. Bracero, Insaf Kouba, Rohit Talwar, Matthew J. Blitz

**Affiliations:** ^1^ Division of Maternal Fetal Medicine, Department of Obstetrics and Gynecology Donald and Barbara Zucker School of Medicine at Hofstra/Northwell Hempstead New York USA; ^2^ Division of Pediatric Cardiology, Department of Pediatrics Donald and Barbara Zucker School of Medicine at Hofstra/Northwell Hempstead New York USA

**Keywords:** cardiovascular fluid dynamics, fetal echocardiography, fetal hypertrophic cardiomyopathy, middle cerebral artery, peak systolic velocity

## Abstract

An elevated middle cerebral artery peak systolic velocity (MCA PSV) in the setting of nondiabetic hypertrophic cardiomyopathy may portend an especially poor prognosis.

## Graphical abstract

Fetal middle cerebral artery peak systolic velocity (MCA PSV) is analyzed to assess fetal anemia. An association between elevated MCA PSV and fetal hypertrophic cardiomyopathy has only been reported once in the literature with the resolution of elevated MCA PSV and normalization of cardiac silhouette after delivery. Two cases are presented with elevated MCA PSV and hypertrophic cardiomyopathy in severely growth‐restricted fetuses resulting in neonatal death. A potential explanation for the relationship involves fluid velocity and dynamics. Elevated fetal MCA PSV in the setting of hypertrophic cardiomyopathy may portend an especially poor prognosis and should be critically studied.

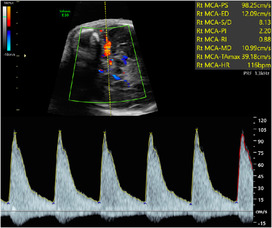

## 1 INTRODUCTION

A fetal middle cerebral artery peak systolic velocity (MCA PSV) greater than 1.5 multiples of the median (MoM) is considered predictive of moderate to severe fetal anemia.^1^ Fetal hypertrophic cardiomyopathy is characterized by hypertrophy of one or both ventricles.^2^ An association between fetal hypertrophic cardiomyopathy and elevated MCA PSV has only been described once previously in a pregnancy, which was complicated by pregestational diabetes mellitus with the resolution of both findings postnatally.^3^ Here we present two cases in the absence of pre‐existing or gestational diabetes with this association and ultimately poor neonatal outcomes.

## 2 CASE PRESENTATIONS

**FIGURE 1:**
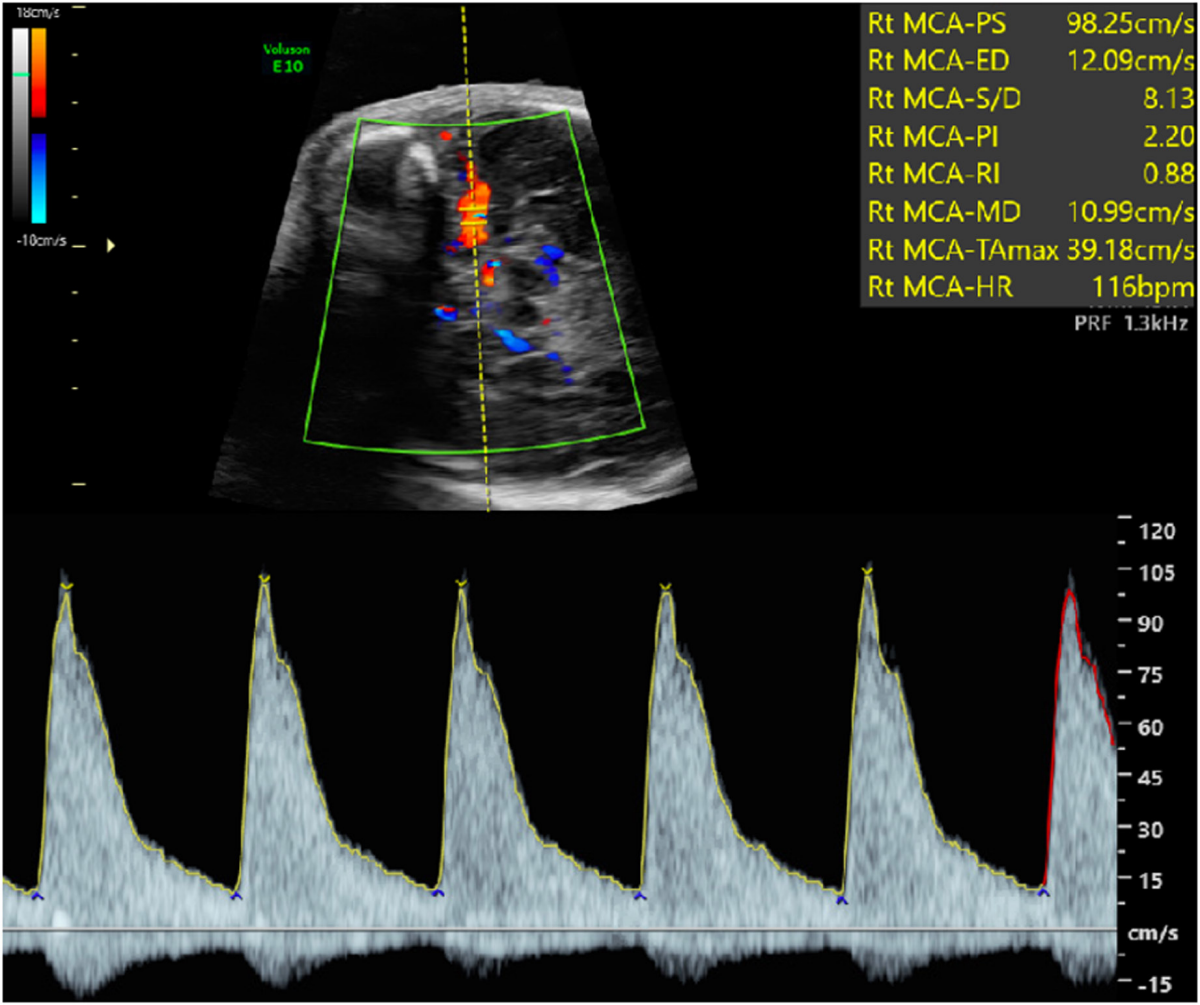
Elevated middle cerebral artery peak systolic velocity (MCA PSV) at >2 MoM at 34 weeks from case 1.

The first case is a 23‐year‐old G3 P1011 with a history of one‐term vaginal birth and one miscarriage. Maternal blood type was O positive, and the antibody screen was negative. First‐trimester ultrasound reported thickened nuchal translucency of 5.3 mm. Chorionic villus sampling was performed. Fetal karyotype was 46, XY (normal male). Chromosomal microarray and Noonan panel were normal. Twenty‐week fetal anatomy ultrasound reported no structural anomalies and the estimated fetal weight (EFW) was at the 5th percentile. A fetal echocardiogram at 21 weeks was normal. At 26 weeks the EFW was at the 1st percentile and the umbilical artery systolic/diastolic ratio was above the 95th percentile. The MCA PSV was elevated at 1.60 MoM. The fetal heart was enlarged. One‐hour glucose challenge test was normal at 130 mg/dL. Parvovirus IgG antibody was positive, and IgM antibody was negative. A fetal echocardiogram performed at 27 weeks of gestation showed an elevated cardiothoracic ratio of 63.5%, moderate concentric left ventricular hypertrophy, moderate right ventricular hypertrophy, no evidence of a left ventricular outflow tract obstruction, normal aortic valve annulus diameter, and no evidence of aortic valve regurgitation. The MCA PSV was 1.65 MoM. At 30 weeks there was increased right ventricular hypertrophy, a mildly dilated right atrium, moderate concentric left ventricular hypertrophy, and a small noncircumferential pericardial effusion. The MCA PSV was 1.65 MoM. At 34 weeks, a small aortic valve annulus measuring 0.50 cm (*z*‐score: −2.39) was noted and the MCA PSV increased to >2.0 MoM (Figure 1).

**FIGURE 2:**
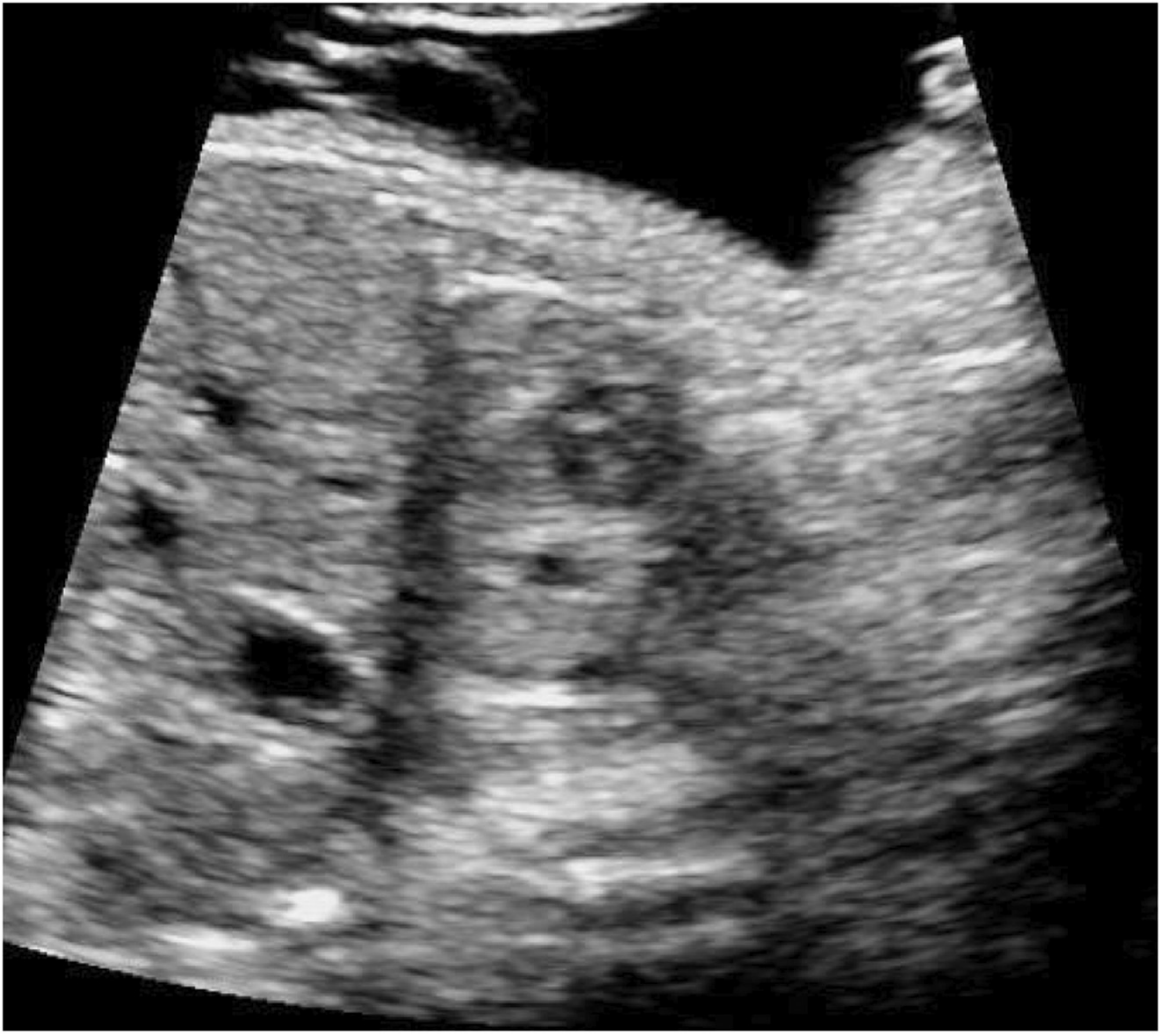
Apical short‐axis view at 34 weeks showing biventricular cardiomyopathy in case 1.

Polyhydramnios and fetal skin edema were noted. Cardiomegaly and biventricular hypertrophy were again seen (Figure 2).

There was no evidence of left ventricular outflow tract obstruction, aortic valve regurgitation, or mitral valve regurgitation. Doppler flow in the umbilical vein, umbilical artery, and ductus venosus was normal. Delivery was recommended. She had a vaginal delivery of a male infant weighing 2210 g with Apgars 1, 1, and 0. The infant expired after 32 min of resuscitation. Postmortem examination revealed skin edema, short long bones, short appearing thorax, and increased nuchal thickness. Umbilical cord arterial blood gas pH was 7.24 and base excess was −4.1 mEq/L. A neonatal hemoglobin level was not available due to coagulation of the neonatal blood.

**FIGURE 3:**
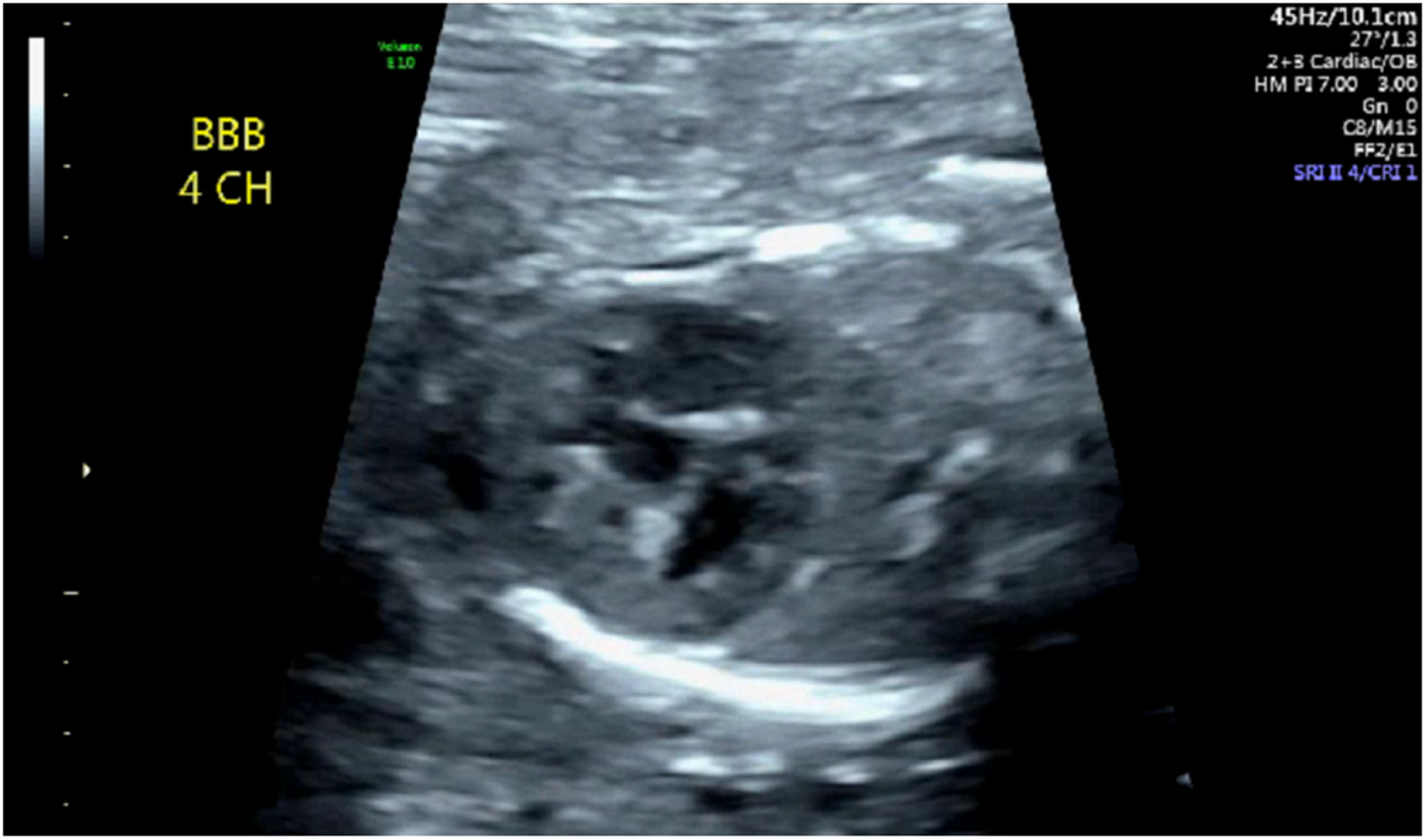
Biventricular hypertrophy at 23 weeks in case 2.

The second case is a 26‐year‐old G1 P0 with dichorionic‐diamniotic twins after intrauterine insemination. Significant medical history included obesity, polycystic ovarian syndrome, and infertility. Maternal blood type was O positive, antibody screen negative, and hemoglobin electrophoresis normal. Cell‐free DNA screening was reported as low risk and both nuchal translucency measurements were normal. At the 20‐week anatomy ultrasound, both fetuses had an EFW less than the 1st percentile. Male twin A had an omphalocele and female twin B had a 2‐vessel umbilical cord and right renal agenesis. Fetal echocardiography at 23 weeks showed that twin B had moderate right ventricular hypertrophy, severe global hypokinesia of the right ventricle, and asymmetric left ventricular hypertrophy of the interventricular septum (Figure 3).

The cardiothoracic ratio was elevated at 59.7% and the MCA PSV was elevated at 1.60 MoM. There was no evidence of left ventricular outflow tract obstruction or aortic valve regurgitation and the aortic valve annulus diameter was normal. No mitral valve regurgitation was seen and Doppler flow in the ductus venosus was normal. Maternal parvovirus IgG and IgM antibodies were negative. One‐hour glucose challenge test was 103 mg/dL. Ultrasound at 24 weeks revealed the fetal demise of twin A. The fetal cardiac findings for twin B at 26 weeks were unchanged. MCA PSV was elevated at 1.69 MoM. Cardiothoracic ratio was 63.3%. Reversed end‐diastolic velocity was visualized in the umbilical artery with normal Doppler flow velocity waveforms in the umbilical vein and ductus venosus. Preterm labor at 27 weeks resulted in a 790‐g male infant with Apgars 1, 4, and 6. The infant required intubation and, despite full resuscitation, the infant expired after 78 min. Umbilical artery blood gas pH was 7.25 and base excess was −1.9 mEq/L. Chest x‐ray revealed diffuse opacification of the lungs. Pathology confirmed a dichorionic‐diamniotic placenta. A neonatal hemoglobin level was not available due to coagulation of the neonatal blood.

## 3 DISCUSSION

The MCA is typically assessed to evaluate for fetal anemia and fetal growth restriction brain sparing. In 2000, Mari and associates illustrated that an increased MCA PSV had 100% sensitivity in predicting moderate/severe fetal anemia.^1^ Mechanistically, likely progressive decreases in fetal hemoglobin cause decreased blood viscosity resulting in elevated MCA PSV.^4^ Severe fetal growth restriction is not known to have an impact on MCA PSV. Doppler evaluation of the MCA has further utility in detecting brain sparing in growth‐restricted fetuses, which causes increased end‐diastolic velocity and decreased MCA pulsatility index.^5^ The MCA PSV has no known association with brain sparing.

The two cases presented suggest a potentially new grim association between elevated MCA PSV and hypertrophic cardiomyopathy. Unfortunately, fetal anemia could not be definitively excluded as coagulation of blood prevented the determination of neonatal hemoglobin levels. Fetal anemia seems unlikely to be the cause of the MCA PSV elevations for multiple reasons. Both mothers had O‐positive blood and ‐negative antibody screens. Neither pregnancy was affected by parvovirus, both mothers had normal hemoglobin electrophoreses, and neither were carriers for thalassemia. There are a few possible explanations for this association. Others have suggested that cardiomyopathy results in enhanced left ventricular contraction with the increased force of blood and likely elevation in MCA PSV.^3^ In addition, the elevated MCA PSV may be related to fluid dynamics. The continuity equation of fluid dynamics states that the mass flow rate into the volume must equal the mass flow rate out of the volume.^6^ Mathematically, this is defined by the following formula:

$${\left(\mathrm{rAv}\right)}_{\text{inlet}}={\left(\mathrm{rAv}\right)}_{\text{outlet}}$$

where *r* = density of fluid; *A* = cross‐sectional area; *v* = flow velocity.

Blood density is constant, so *v*_outlet_ = *v*_inlet_ × (*A*_inlet_/*A*_outlet_). This means blood velocity increases as blood passes through a narrowed area. In case 1, this narrowed area may be the aortic valve annulus and in case 2 it may be the asymmetric left ventricular hypertrophy. The blood may then continue from the aorta through the brachiocephalic trunk to the common carotid artery through the internal carotid artery and into the middle cerebral artery detected as increased MCA PSV. Either mechanism may be correct, or it may be a combination of both.

Regardless of the mechanism, primary hypertrophic cardiomyopathy with an elevated MCA PSV in nondiabetic pregnancies may be an ultrasonographic sign portending an especially poor prognosis. It is known that primary fetal hypertrophic cardiomyopathy diagnosed prenatally generally is associated with poor outcomes.^7^ One retrospective review at a single center from 2000 to 2012 found a transplantation‐free survival from diagnosis to 1 month and 1 year of life to be 35% and 18%, respectively.^8^ To our knowledge, the association between hypertrophic cardiomyopathy and elevated MCA PSV has only been described once in the literature^3^ with a cause of the hypertrophic cardiomyopathy identified. Specifically, that case was complicated by pre‐existing diabetes mellitus, with an ultimately favorable outcome. Further, the MCA PSV normalized postnatally and the fetal cardiac interventricular septum, which was thickened to 10 mm prenatally was reduced to 6 mm at 10 days of life. Our cases were not complicated by diabetes and both infants did poorly.

## 4 CONCLUSION

In summary, our two cases suggest that an elevated MCA PSV in the setting of nondiabetic hypertrophic cardiomyopathy may portend an especially poor prognosis. Future research may focus on this relationship, mechanism, and potential utility in clinical practice. The MCA PSV may have further clinical value than mere identification of cases of fetal anemia.

## FUNDING STATEMENT

This study did not receive any specific grant from funding agencies in the public, commercial, or not‐for‐profit sectors.

## ETHICS APPROVAL STATEMENT

Written informed consent for this report was obtained.

## PATIENT CONSENT STATEMENT

Written informed consent for this report was obtained.

## AUTHOR CONTRIBUTIONS

**Nathan A. Keller:** Writing – original draft; writing – review and editing. **Luis A. Bracero:** Writing – original draft; writing – review and editing. **Insaf Kouba:** Writing – review and editing. **Rohit Talwar:** Writing – review and editing. **Matthew Blitz:** Writing – review and editing.

## CONFLICT OF INTEREST STATEMENT

The authors report no conflicts of interest.

## Data Availability

Data sharing not applicable to this article as no datasets were generated or analysed during the current study.
